# Sulfide dynamics at the gut-microbiota interface: diet, oxygen and redox interplay

**DOI:** 10.1080/19490976.2026.2681720

**Published:** 2026-06-02

**Authors:** Roshan Kumar, Ruma Banerjee

**Affiliations:** a Departments of Biological Chemistry, Michigan Medicine, University of Michigan, Ann Arbor, MI, USA

**Keywords:** Hydrogen sulfide, hypoxia, redox, energy metabolism, colon

## Abstract

Teeming with microbes, the unique biogeography of the gut is shaped by interactions between diet, host and microbial metabolism. Hydrogen sulfide represents one such plane of interaction in the lower gut where it is largely the product of microbial activity. Sulfide oxidation by host epithelial cells helps shape a severely hypoxic luminal environment in which obligate anaerobes thrive and furnish among other products, butyrate, a fuel of choice for colonocytes. This metabolic symbiosis in healthy gut is supported by diet, and disrupted when the host sulfide oxidation capacity is exceeded, with resultant local and long-range impacts, including increased susceptibility to enteric pathogens and behavioral changes. Under homeostatic conditions, sulfide oxidation tunes host energy and redox metabolism that is corrupted under dysbiosis linked to gastrointestinal diseases. H_2_S could also be important for inducing a metabolic state change as in hibernating animals, by increasing energy storage in the form of reduced cofactors as well as increasing intracellular oxygen. In this review, we bracket luminal free sulfide exposure to colonocytes based on bioenergetic studies on colon-derived cells, discuss the microbial pathways for sulfide generation, and their interplay with dietary sulfur and host oxygen and redox metabolism.

## Gut sulfide conversations

**Figure 1.**
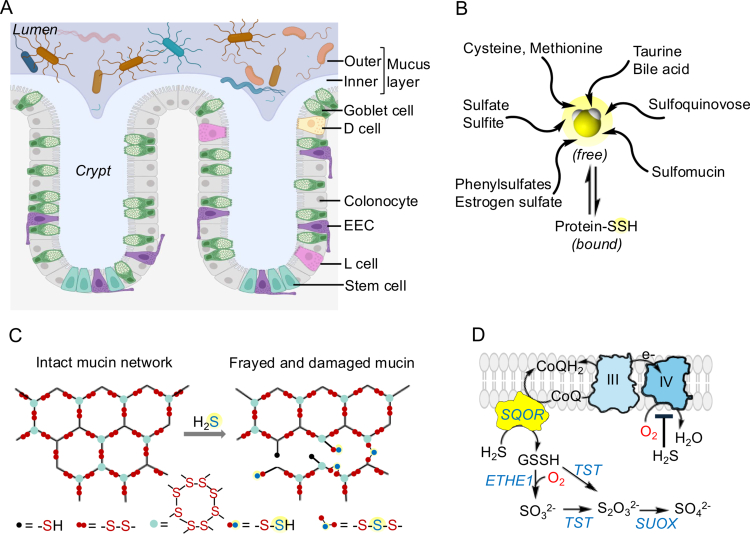
Architecture of the colon-microbiota interface and sources of H_**2**_S. (A) Crypts are protected by two layers of mucin: a tightly packed inner layer that restricts microbial entry and a loose outer layer that is a source of nutrients to resident microbes. The epithelial layer is a composite of enterocytes and specialized cells with stem cells residing at crypt bases. EEC is enteroendocrine cell. This panel was created using BioRender. (B) Overview of organic and inorganic sulfur sources for microbial H_2_S production. (C) The polymeric mucin (Muc2) network is formed via disulfide-linked dimers and trimers, which are susceptible to nucleophilic attack by sulfide anions. In healthy gut, mucin likely serves as a sulfide sink, contributing to the bound sulfane sulfur pool and dysbiosis could lead to excessive structural damage. For clarity, attack of sulfide is not shown at the trimer interface. (D) The sulfide oxidation pathway in host cells starts with the SQOR-catalyzed oxidation of H_2_S that is coupled to O_2_ reduction by complex IV via the intermediate electron carrier, CoQ. The remainder of the pathway involves oxidations (catalyzed by ETHE1 and SUOX) and sulfur transferase reactions catalyzed by TST. Only the major sulfur species are shown and the reactions are not balanced.

The close physical proximity, sheer density, and diversity of the microbial community to colon has profound implications for human health and disease risk.^1^ ^,^ ^2^ A monolayer of columnar epithelial cells interspersed with specialized ones, collectively execute the absorptive and secretory functions of the lower gut (Figure 1A).^3^ A thick mucus layer secreted by goblet cells, forms the physical interface between host and microbiota, and is organized into a densely packed, adherent inner layer that is largely microbe-free, and a loosely packed, nonadherent outer layer that both houses and nourishes microbes.^4^ MUC2, a netlike polymer that is extensively crosslinked via disulfides and is heavily glycosylated, is the most abundant mucin in colon.^5^ The mucus proteome comprises ~50 proteins, the functions of most of which await characterization.^4^ ^,^ ^5^ Physical and chemical heterogeneity, contributed by factors such as osmolality, O_2_ levels and pH, shape the dynamic ecosystem of the colon that teems with microbes.^6^ The genetically tractable commensal, *Bacteroides thetaiotaomicron,* has recently been engineered for noninvasive reporting of increased osmolality that is associated with clinical malabsorption.^7^ Together with advances in spatial single cell transcriptional analysis,^8-12^ these innovations represent powerful approaches for charting gut bio-cartography, which is largely unknown.

Commensality in the gut is brokered via the boisterous exchange of chemical signals and nutrients across the mucosal divide. Hydrogen sulfide (H_2_S) is one such molecular signal that, being highly soluble and membrane permeable,^13^ has the potential to exert both local and long-range effects. While better known for its notoriety as a genotoxin,^14^ respiratory poison and damaging to mucosal integrity and barrier function,^15^ recent studies are beginning to reveal the beneficial effects of H_2_S that are choreographed via the intricate interplay of host and microbial metabolism.

Very high luminal H_2_S concentrations have been reported in human colon (1.0-2.4 mM)^16^ and fecal samples (0.2-3.4 mM).^17-19^ Similarly high concentrations have also been reported in rat cecum (1.5 mM)^20^ and in the lumen of mouse large intestine (0.2-1.0 mM).^21^ Importantly, these values represent total sulfide concentrations (i.e. protein-bound and free) and are very likely overestimated due to a common technical artifact associated with sample preparation, i.e. the use of acidic conditions that liberate sulfide from iron-sulfur containing proteins.^22^ ^,^ ^23^ Based on reports that only 1-8% of the total sulfide in fecal samples is in the free form,^19^ ^,^ ^20^ we estimate that the relevant free sulfide concentration is 10-190 µM in human colon lumen, and within a similar 2-120 µM range in rodents. Sulfur intake (dietary animal versus plant protein, taurine, sulfoquinovose) and supplements (e.g. chondroitin sulfate) as well as metals like iron, zinc and bismuth, which bind free sulfide,^24^ influence gut H_2_S levels (Figure 1B). The three-way interaction between diet, microbial and host sulfide metabolism is largely unexplored and ripe for investigation. In this review, we focus on H_2_S homeostasis and its interplay with O_2_ and redox metabolism. This understudied intersection shapes microbiome demographics, is shaped by diet, and, in turn, impacts host health and behavior.

## Mucin modulates host sulfide exposure

The concentration window within which sulfide exerts its beneficial effects is an important consideration for studying colon physiology. H_2_S not only has the potential to inhibit complex IV (or cytochrome c oxidase) in the electron transport chain, but also ACADS,^25^ ^,^ ^26^ which converts butyryl-CoA to crotonyl-CoA. Colonocytes preferentially utilize butyrate^27^ generated by the microbial fermentation of complex carbohydrate and fiber remnants, creating a dependence on oxidative phosphorylation. The disulfide-rich mucus layer presents a chemical barrier that attenuates sulfide exposure of colonocytes to luminal sulfide (Figure 1C). Cysteines comprise ~10% of Muc2, and intermolecular disulfide bonds are integral to its extensive polymeric network structure.^4^

A recent study connected heme-induced colon epithelial damage and hyperproliferation to mucolysis, i.e. addition of sulfide to disulfide bonds, leading to the formation of persulfides and trisulfides and possibly, other catenated sulfur species (Figure 1C).^15^ The ensuing structural degradation and loss of viscoelasticity increased access to mucin-degrading *Akkermansia.* This mechanism of mucus barrier degradation by a combination of sulfate-reducing and mucin-degrading bacteria with loss of host epithelial protection, might be important in the etiology of inflammatory bowel diseases.^28^ In healthy gut, some of the sulfide produced by gut-resident bacteria is undoubtedly tied up in the mucus layer, which likely serves as a sulfide sponge and decreases host exposure to the gas.

## Sulfide oxidation can power and poison the electron transport chain

Cysteine and homocysteine are the primary sulfur sources for host sulfide synthesis, which is catalyzed by enzymes in the transsulfuration (CBS and CTH) and cysteine catabolism (MPST) pathways, and has been reviewed.^29^ ^,^ ^30^ Microbial metabolism is however, the major contributor to gut H_2_S,^31-33^ which is consistent with low colon expression of all three sulfide-generating enzymes as reported in the Human Protein Atlas.

Sulfide quinone oxidoreductase (SQOR), along with ETHE1, TST and SUOX comprise the H_2_S oxidation machinery (Figure 1D).^34-37^ Host colon epithelial cells expresses high levels of SQOR, a mitochondrial inner membrane-anchored enzyme that catalyzes oxidative sulfur transfer from H_2_S to glutathione, forming glutathione persulfide.^38^ ^,^ ^39^ Electrons released during H_2_S oxidation move to coenzyme Q (CoQ) and then, into the electron transport chain (ETC). Sulfide oxidation therefore consumes O_2_ and generates ATP.^40^ Human SQOR has an uncommon cysteine trisulfide cofactor; H_2_S activates its installation and increases enzyme activity 2- to 5-fold.^26^ ^,^ ^41^ ^,^ ^42^ The promiscuity of SQOR allows alternate acceptors like cysteine, homocysteine, sulfite and methanethiol, a volatile organic sulfur compound produced by microbiota, to function as sulfane sulfur acceptors.^43^ The 2-fold higher solubility of H_2_S in lipid membranes versus water,^13^ contributes to its high rate of reaction with SQOR (estimated to be 3.0 × 10^7^ M^−1^s^−1^ at 37°C).^39^ The cellular capacity to clear H_2_S depends on SQOR expression levels, which vary widely across tissues, as well as on CoQ and O_2_ concentrations. Importantly, there are two O_2_-consuming steps in the sulfide oxidation pathway (Figure 1D). One mole of O_2_ is used by the dioxygenase ETHE1, while half a mole of O_2_ is consumed at complex IV, per mole of H_2_S oxidized. While varied physiological effects have been ascribed to H_2_S,^44^ it is possible that many of them are modulated via its oxidation e.g. its effects on neoangiogenesis^45^ and on shaping gut biogeography, as described below.

## Microbial pathways for H_2_S biogenesis

The two major routes for microbial H_2_S biogenesis in gut originate from sulfate assimilation and cysteine catabolism. While H_2_S is generated via both dissimilatory and assimilatory sulfate reduction, it is the final product in the former, and an intermediate in cysteine synthesis in the latter pathway. Sources of sulfate that are available to gut microbes include: diet (dried fruits, nuts, cruciferous vegetables, fermented beverages are rich sources),^46^ supplements like chondroitin sulfate,^47^ organosulfur compounds like sulfomucins in the gut lining,^48^ and metabolites like sulfoquinovose (6-deoxy-6-sulfoglucose),^49^ taurine and taurocholic acid (Figure 1B). A survey of cysteine, taurine, sulfite/sulfate metabolic pathways associated with H_2_S production across 514 fecal microbial genomes from the Human Microbiome Project, revealed the presence of sulfidogenic genes across six phyla (Proteobacteria, Firmicutes, Bacteroidetes, Fusobacteria, Actinobacteria, and Synergistetes). Not surprisingly, cysteine-dependent genes were the most abundant; as discussed below, H_2_S generation is the canonical reaction catalyzed by only a subset of the encoded enzymes within this group.^50^ Metagenomics analyzes point to the existence of syntrophy or mutualistic feeding relationships amongst sulfidogenic bacteria for the complete degradation of organosulfur compounds.^50^ In the following subsections, major pathways leading to gut microbial H_2_S generation are discussed briefly.

### Dissimilatory sulfate reduction

**Figure 2.**
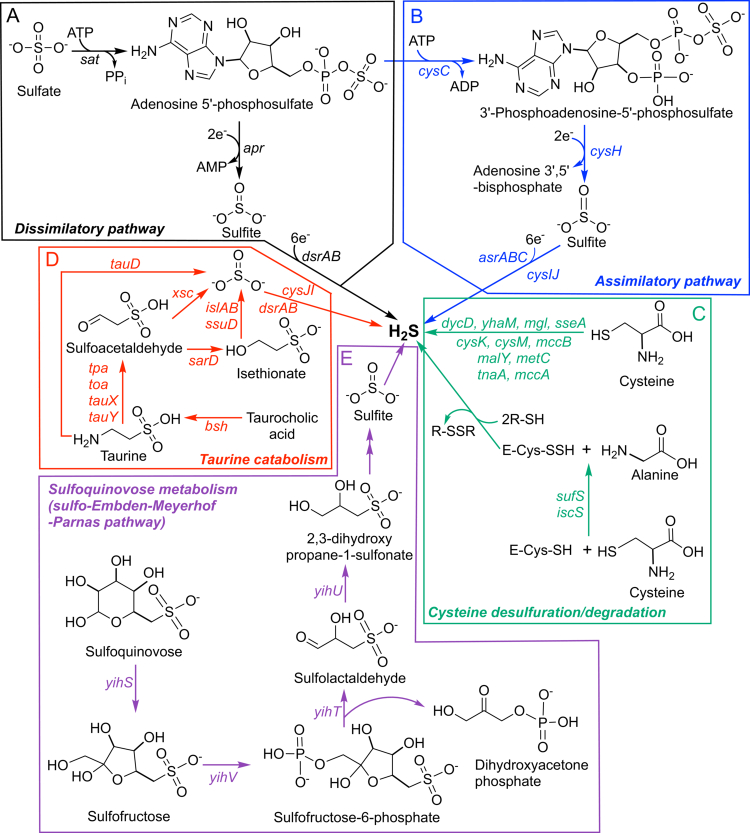
Scheme showing microbial H_2_S production pathways. H_2_S can be generated via dissimilatory (A) or assimilatory (B) sulfate reduction, (C) cysteine desulfuration, (D) taurine catabolism, or (E) sulfoquinovose catabolism. RSH in (C) represents a physiological thiophilic acceptor. The reactions are not balanced and the gene names for the encoded enzymes are in italics.

Sulfate-reducing bacteria are obligate anaerobes that are highly prevalent but not highly abundant in human gut. The most common representatives of the *Desulfobacterota* phylum (reclassified from *Deltaproteobacteria*) that contribute to intestinal sulfate reduction to H_2_S are *Desulfovibrio* spp and *Bilophila wadsworthia*.^51^ Other genera harboring the capacity for dissimilatory sulfate reduction in the human gut include *Collinsella*, *Eggerthella, Enterococcus*, *Flavinofracter*, *Gordonibacter*, and *Roseburia*.^50^ Sulfate-reducing bacteria use sulfate as the terminal electron acceptor, and in the environment, can derive electrons from the oxidation of hydrogen or organic compounds such as lactate, acetate or butyrate.^52^ The 8-electron reduction of sulfate to H_2_S via the dissimilatory sulfite reductase pathway proceeds via: (i) ATP-dependent activation of sulfate to form adenosine-5′-phosphosulfate catalyzed by ATP sulfurylase, (ii) reduction of adenosine-5′-phosphosulfate by APS reductase to sulfite with concomitant elimination of AMP, and (iii) sulfite reduction catalyzed by dissimilatory sulfite reductase, DsrAB (Figure 2A). DsrAB is a heterodimeric enzyme with a siroheme-coupled [4Fe-4S] cluster. Sulfite reduction occurs via an enzyme-bound trisulfide intermediate on a different protein, DsrC. In the final step, electrons for the reductive elimination of H_2_S from the trisulfide on DsrC are donated by menaquinol via the membrane-associated DsrMKJOP complex, and is coupled to energy-conserving proton translocation.^53^

### Assimilatory sulfate reduction

The capacity to incorporate sulfate into organic sulfur compounds via assimilatory sulfate reduction is widespread among microbes.^50^ ^,^ ^54^ Several gut microbes, including sulfate-reducing bacteria, harbor the assimilatory sulfate reductase pathway and generate H_2_S as a biosynthetic intermediate primarily for cysteine synthesis. In contrast to the dissimilatory pathway, assimilatory sulfate reduction is not linked to respiration and energy conservation. Adenosine-5′-phosphosulfate is phosphorylated further in the assimilatory pathway by a kinase, forming 3’-phosphoadenosine-5’-phosphosulfate, which upon reduction, forms sulfite and adenosine 3’, 5′-bisphosphate. In the final step, sulfite is reduced to sulfide by the AsrABC complex (Figure 2B).^55^ The AsrABC complex shares significant structural and mechanistic similarity with DsrAB, including a siroheme-[4Fe-4S] catalytic center, reflecting a common evolutionary origin.^56^ Bacteria across five phyla (Actinobacteria, Firmicutes, Fusobacteria, Spirochetes and Bacteriodetes) harbor the *asrABC* genes and include the common gut microbiome residents, *Fusobacterium nucleatum* and *Clostridium intestinale*.^50^ ^,^ ^57^

### Cysteine degradation

Cysteine desulfurases are pyridoxal 5’-phosphate-dependent enzymes that catalyze the elimination of sulfur from cysteine, generating alanine and an enzyme-bound persulfide intermediate (Figure 2C).^58^ The enzyme-bound sulfane sulfur is transferred to acceptor molecules in pathways leading to the biosynthesis of iron-sulfur clusters, tRNA thiolation, and cofactors such as thiamin and biotin.^58^ A bioinformatic analysis-based curation of cysteine degrading enzymes in the human gut microbiome classified the H_2_S-generating reactions as: (i) primary, (*dcyD, mgl sseA, yhaM*), (ii) secondary i.e. where H_2_S synthesis is the non-canonical function (*cysK, cysM, malY, metC, mccB*), and (iii) erroneous (*iscS, mccA and tnaA*) (Figure 2C).^59^ The analysis led to the conclusion that the capacity for H_2_S production via cysteine catabolism is ubiquitous in the human gut microbiome and distributed across 13 phyla and 141 genera,^50^ although experimental validation and quantitative evaluation of this activity remains to be furnished.

### Taurine metabolism

Examples of sulfonates that are catabolized by gut microbes include taurine-conjugated bile acids, taurine, an abundant amino acid, and isethionate. Microbial bile salt hydrolases (*bsh*) hydrolyze taurine-conjugated bile salts, which are derived from cholesterol, releasing taurine (Figure 2D).^60^ Within the anaerobic colonic environment, multiple bacterial taxa utilize distinct desulfonation strategies to catabolize taurine, producing sulfite that can be further reduced to H_2_S. In the canonical pathway, taurine-pyruvate aminotransferase (*tpa*) converts taurine to sulfoacetaldehyde, which is then metabolized to yield acetyl-CoA and sulfite (Figure 2D).^61^ ^,^ ^62^ In addition to this route, intestinal bacteria like *Bilophila wadsworthia* harbor a glycyl radical enzyme-dependent pathway that operates exclusively under anoxic conditions and catalyzes direct C-S bond cleavage in taurine.^63^ The pathway involves taurine-pyruvate aminotransferase-mediated formation of sulfoacetaldehyde, which is reduced by sulfoacetaldehyde reductase (*sarD*) to isethionate, followed by cleavage to sulfite and acetaldehyde catalyzed by isethionate sulfite lyase (*islA*) (Figure 2D). Sulfite generated through this pathway serves as an efficient substrate for microbial reductases, positioning taurine as a major organic sulfur source for colonic H_2_S production. Taurine can also be oxidized to sulfite in a reaction catalyzed by taurine dioxygenase (*tauD*) found in facultative gut bacteria (*Escherichia*, *Enterobacter*, *Citrobacter*, *Morganella*, *Hafnia*, and *Raoultella*). Lacking the genes encoding either assimilatory or dissimilatory sulfite reductase, these organisms likely use taurine for assimilation rather than H_2_S biogenesis.^50^

### Sulfoquinovose metabolism

**Figure 3.**
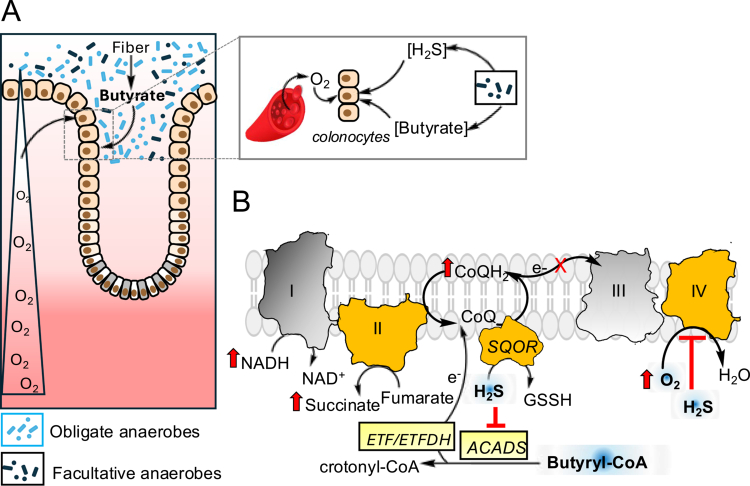
Metabolic host-microbial symbiosis shapes the luminal environment. (A) In healthy gut, epithelial metabolism is key to maintaining a steep O_2_ gradient. Butyrate and H_2_S derived from gut microbes drive high O_2_ metabolism in colonocytes that are perfused with O_2_ from the vasculature (*inset*). Colonocyte utilization of gut-derived metabolites in turn, drives luminal hypoxia (B). Bioenergetic and redox costs of surpassing epithelial sulfide oxidation capacity. An increase in H_2_S inhibits complex IV, promotes fumarate-dependent CoQH_2_ recycling by complex II, inhibits ACADS-dependent oxidation of butyrate, increases local O_2_ and induces a reductive shift in the redox cofactor pools.

Sulfoquinovosyl diacylglycerol is a sulfonolipid that is abundant in green vegetables, algae and many other photosynthetic organisms.^64^ Its polar head group comprises sulfoquinovose or sulfonated glucose, which is abundant in the global sulfur cycle and approximately equivalent to the organosulfur reservoir represented by protein cysteine and methionine.^57^ Multiple pathways have been described for sulfoquinovose catabolism following its release from the sulfonolipid via a bacterially encoded sulfoquinovosidase. In the sulfo-Embden-Meyerhof-Parnas pathway, sulfoquinovose is converted in four steps to dihydroxyacetone phosphate that is processed via central carbon metabolism for energy generation, and either 2,3-dihydroxypropane 1-sulfonate (during aerobic growth) (Figure 2E) or 3-sulfolactate (during anaerobic growth).^65^ ^,^ ^66^ This pathway is widespread in Enterobacteriaceae and interspecies cross-feeding is required to complete the degradation of 2,3-dihydroxypropane 1-sulfonate, which can be cleaved to sulfite and reduced to H_2_S.^67^ Alternatively, sulfoquinovose can undergo oxygenolytic C-S bond cleavage to sulfite.^68^ The host lacks the ability to metabolize sulfoquinovose, which is instead a substrate for few, albeit abundant, gut microbes including *B. wadsworthia* and *Agathobacter rectalis* (previously *Eubacterium rectale*).^49^ It has been reported recently that unlike human microbiota, conventional laboratory mice are unable to degrade 2,3-dihydroxypropane 1-sulfonate, limiting their utility for the study of the prebiotic, sulfoquinovose.^67^

## Symbiotic H_2_S-O_2_-butyrate metabolism shapes gut bioenvironment

A steep radial O_2_ gradient runs across the colon, ranging from a virtually anoxic lumen to a vascular, O_2_-perfused subepithelial mucosa (Figure 3A, *left*).^69^ Colonocytes reside in a liminal zone within this gradient and exhibit a high O_2_ demand by virtue of their butyrate and sulfide consumption. H_2_S and butyrate metabolism intersect at the CoQ pool in the ETC and rely on O_2_ as a terminal electron acceptor (Figure 3B). Unlike SQOR, which reduces CoQ directly as it oxidizes H_2_S, ACADS transfers electrons released from butyrate oxidation to CoQ via a 2-protein wire, comprising ETF and ETFDH (Figure 3B). The dynamic interplay of H_2_S and butyrate metabolism is a critical factor in governing luminal hypoxia^70-72^ and shaping microbial composition in healthy gut.^73-75^ The dominance of obligate anaerobes in turn, supports butyrate^76^ ^,^ ^77^ and sulfide production (Figure 3A, *inset*).

Epithelial butyrate consumption is also a critical driver of the diminishing butyrate gradient along the crypt length, which protects progenitor stem cells at the base from its antiproliferative effects.^78^ In contrast to colonocytes, stem cells rely on aerobic glycolysis to sustain a rapid proliferation rate.^79^ A dysbiotic shift that is permissive for the expansion of facultative anaerobes, is correlated with intestinal inflammation, antibiotic treatment and chronic illnesses.^73^ ^,^ ^80-82^

Unlike other cell types that also rely primarily on oxidative phosphorylation, e.g. M2 macrophages,^74^ only colonocytes are characterized by profound hypoxia, implicating an underlying cell-specific adaptation that was recently identified to be H_2_S oxidation.^83^ In this study, epithelial hypoxia, visualized with a nitroimidazole dye, was shown to colocalize with immunohistochemical staining for SQOR expression in murine colon but not in the small intestine. In comparison to the large intestine, SQOR expression is low in the small intestine, which is also a less hypoxic compartment. Restricting sulfide bioavailability with bismuth citrate supplementation, significantly decreased epithelial hypoxia, as did knocking out SQOR in intestinal epithelial cells (SQOR^ΔIEC^ mice).^83^ Collectively, these data provide compelling evidence for sulfide oxidation being a primary driver of epithelial hypoxia. Remarkably, loss of H_2_S oxidation capacity in the intestinal epithelium increased facultative anaerobes like *Bacilli* and *Enterobacterales* and microaerophilic bacteria belonging to *Helicobacteraceae.* In contrast, the obligate anaerobe *Clostridia,* including the *Lachnopirales* taxon, a butyrate producer, decreased in abundance, which was correlated with lower cecal butyrate.^83^ SQOR^ΔIEC^ mice showed a 3-fold increase in pathogen burden compared to littermate controls following infection with *Salmonella enterica* serovar Typhimurium, as well as greater pathogen dissemination to liver and kidney together with higher levels of the inflammatory marker, calprotectin-2.^83^

Bismuth subsalicylate (BSS, commonly known as Pepto-Bismol) is a common over-the-counter medication to treat GI distress but also traps H_2_S as insoluble bismuth sulfide.^84^ In a study on human subjects, BSS elicited profound changes in the microbiome with an expansion of potentially pathogenic Pseudomonadota.^85^ BSS use was also associated with increased inflammation, decreased T cell activation and Th1 collapse. In the same study, BSS administration increased susceptibility of mice to the enteric pathogen, *Salmonella*.

Based on these studies, a model of metabolic symbiosis is emerging in which microbially-derived sulfide is oxidized by epithelial cells, driving luminal hypoxia. The latter creates conditions in which obligate anaerobes that produce butyrate thrive, and furnish colonocytes with a choice fuel source, while simultaneously contributing to high O_2_ consumption via *β*-oxidation (Figure 3A, *inset*). Importantly, the emerging picture is that microbial sulfide biogenesis and host sulfide oxidation are beneficial to gut health, and confer colonization resistance to enteric pathogens.^83^ ^,^ ^85^

## Estimated colonocyte exposure to microbial H_2_S

The actual concentration and type of exposure, i.e. acute or chronic, that colonocytes typically experience, is unknown, but highly pertinent to evaluating how microbial H_2_S impacts host metabolism. In this context, the concentration window in which H_2_S switches from having a stimulatory to a net inhibitory effect on respiration in colon-derived cells might be insightful. At low concentrations (5-20 µM), bolus administration of H_2_S to HT-29 cells stimulates O_2_ consumption, while higher concentrations (≥30 µM) induce net inhibition^86^ ^,^ ^87^ with effects on mitochondrial bioenergetics that persist for 24-48 h.^88^ Thus, while HT-29 cells clear a 20 µM bolus of H_2_S and regain ~90% of the initial respiratory rate in 5 min,^88^ chronic exposure to the same concentration over 24 h, results in a 90% decrease in the respiratory rate and recovery of the basal O_2_ consumption rate over 48 h.^89^ Cell viability is however, unaffected (up to 125 µM H_2_S, 24 h exposure), as cells emerge from S-phase arrest and resume progression through the cell cycle upon sulfide withdrawal.^89^ ^,^ ^90^ These *in vitro* analyzes suggest that the beneficial effects of acute exposure in colonocytes occur at < 20 µM H_2_S, while chronic exposure must be << 10 µM to preserve *β*-oxidation-based energy metabolism in healthy gut. Importantly, our estimates are significantly lower than the millimolar total sulfide concentrations discussed above, which are generally referenced in the context of luminal H_2_S.

## Microbial H_2_S tunes host O_2_ and redox metabolism

**Figure 4.**
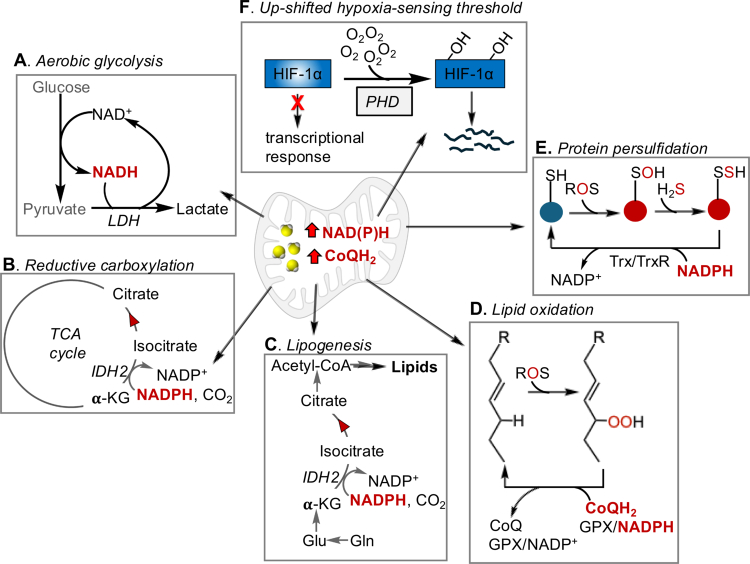
Metabolic shifts due to H_2_S-induced inhibition of mitochondrial O_2_ consumption. H_2_S-induced reductive shift in the NAD(*P*)^+^ and CoQ pools results in: (A) increased aerobic glycolysis, (B) IDH-catalyzed reductive carboxylation, (C) increased lipogenesis using glutamine as a carbon source, (D) increased lipid peroxidation, (E) increased protein persulfidation, and (F) an upshift in the threshold for hypoxia sensing by PHD.

Even partial inhibition of O_2_-dependent H_2_S oxidation has manifold cellular effects, with implications for redox metabolic regulation. Importantly, a second route with complex II operating in reverse and fumarate serving as the terminal electron acceptor, allows cells to prioritize H_2_S clearance when O_2_-dependent oxidation via complex IV is inhibited (Figure 3B).^91^ The relative importance of the alternative path for H_2_S oxidation is not known, and will be governed by the concentration of fumarate as well as the proximity of SQOR to complex II versus complex III, which often localizes in supercomplexes.^92^ Decreased electron flux to complex IV induces a reductive shift in the CoQ^93^ and NAD^94^ cofactor pools with metabolic ramifications that ripple through redox active nodes^95^ ^,^ ^96^ (Figure 4). Decreased electron flux redirects energy metabolism from oxidative phosphorylation toward glycolysis and SQOR knockdown further sensitizes cells to this bioenergetic shift^94^ (Figure 4A). Expansion of the NAD(*P*)H pool drives IDH-dependent reductive carboxylation, concomitantly converting glutamine carbon to lipids that are stored in lipid droplets^86^ ^,^ ^97^ ^,^ ^98^ (Figures 4B,C). A reductive shift in the cofactor pools also has the potential to increase reactive oxygen species (ROS) that enhances lipid peroxidation^99^ and cysteine oxidative modifications including persulfidation^100-102^ (Figures 4D,E). Since both types of early modifications are reversed by redox systems that depend on NAD(*P*)H or CoQH_2_ for reducing equivalents (as shown in Figures 4D,E), the magnitude of the oxidative shift would depend on the duration of sulfide exposure, with chronic exposure likely favoring repair and possibly, a hyper-reduced proteome.

Complex IV is the major O_2_ consumer in actively respiring cells and its inhibition by sulfide increases intracellular O_2_ and activates PHD, the principal O_2_ sensor, which hydroxylates HIF, consigning it to proteasomal degradation^99^ (Figure 4F). Thus, by inhibiting complex IV, sulfide can shift the intracellular threshold for hypoxia sensing even under conditions of ambient hypoxia. An-H_2_S induced reductive shift in the CoQ pool also affects other CoQ users including DHOD, G3PDH, PROD and ETFDH, impacting pyrimidine, lipid, and amino acid metabolism.

H_2_S can reversibly induce a state of suspended animation, associated with a profound lowering of the metabolic rate and core body temperature.^103^ Preconditioning mice with a 20 min exposure to H_2_S, extended survival under lethal hypoxia (5% O_2_) from 20 min to >6.5 h without apparent adverse effects.^104^ We speculate that H_2_S-based regulation of the cellular O_2_ economy and promotion of energy storage in the form of reduced cofactors, might be relevant to hibernation physiology in addition to being important for tuning redox metabolism in non-hibernating animals. The contributions of microbial versus host H_2_S to hibernation behavior is unknown, and would be interesting to dissect in a model organism like the arctic ground squirrel, which exhibits extreme physiological adaptations for surviving an 8-9 month long hibernation period.^105^

## Local, long-range and dysbiotic effects of diet-genotype-sulfide metabolism interactions

Hereditary SQOR deficiency is a rare autosomal recessive disease that presents with the neurological symptoms of Leigh disease.^106^ Since an estimated ~70% of systemic sulfide metabolism can be traced to gut H_2_S metabolism,^91^ it is pertinent to ask whether dysbiotic gut sulfide metabolism contributes to the neurological and other presentations of SQOR deficiency. In addition to microbial composition, gut sulfide metabolism is modulated by diet, increasing with a higher dietary protein intake.^18^ In healthy gut, high SQOR expression shields colonocytes from the detrimental effects of H_2_S on cellular bioenergetics, mitochondrial architecture and redox metabolism. While H_2_S is challenging to measure in tissue, it leaves behind fingerprints when its levels are elevated, including loss of the MT-CO1 (or COX1) subunit of complex IV, a pronounced reductive shift in the CoQ pool, and increased serum and urine thiosulfate.^32^ The first two of these changes are prominently featured in colon of SQOR^ΔIEC^ mice but absent when the mice are treated with broad spectrum antibiotics. These data suggest that either loss of SQOR or exceeding host H_2_S oxidation capacity, impairs oxidative phosphorylation, with metabolic effects rippling through redox nodes (Figure 4). Additionally, loss of gut epithelial SQOR sensitize mice to a 2.5-fold increase in dietary methionine (i.e. the difference in animal versus plant protein), inducing crypt degeneration, decreased goblet cell density, and thinning of the mucus barrier.^32^

Long-range effects in SQOR^ΔIEC^ mice maintained on a 2.5x methionine diet are visible in liver and brain, i.e. the signature loss in MT-CO1 and a reductive shift in the CoQ pool, albeit of a lower magnitude than seen in gut.^32^ Further, increased ketone bodies in serum indicate elevated liver ketogenesis. Remarkably, these mice exhibit markedly lower exploratory locomotor behavior although the molecular basis of this change remains to be identified.^32^ Long-range effects of dietary sulfur amino acids have also been reported in a murine model of chronic kidney disease.^107^ In this model, high methionine- and cysteine-containing diet increased cecal sulfide, decreased microbial production of the uremic toxins, indole and indoxyl sulfate, and ameliorated disease progression, which was ascribed to persulfidation of microbial tryptophanase.^107^

Associations, more positive than negative, between H_2_S and inflammatory bowel diseases and colorectal cancer have been reported.^108-111^ Dietary changes can rapidly alter the human gut microbiome; an increase in the sulfite-reducing *Bilophila wadsworthia* and decrease in Firmicutes, which metabolize plant polysaccharides, are associated with an animal-based diet.^112^ A “sulfur microbial diet” characterized by high intake of red meats and low-calorie beverages combined with low intake of vegetables and fruits, has been associated with increased risk for both Gl (colorectal cancer) and non GI (obesity, nonalcoholic fatty liver disease and all-cause mortality) diseases.^110^ ^,^ ^113-115^ A plasma metabolite signature associated with the “sulfur microbial diet” includes changes in bile acids, lipids and amino acids derivatives.^116^ Our current understanding on the interactions between diet, microbiome and host sulfur metabolic interactions represents just the tip of an iceberg of knowledge that awaits discovery with implications for normal physiology and behavior as well as both GI and non GI diseases.

## Open questions

- What is the magnitude and nature (acute versus chronic) of gut microbial sulfide exposure in healthy gut, and how do dietary changes affect these dynamics?
- Are molecules other than H_2_S involved in relaying changes in gut metabolism both locally and distally?
- Can innovative technologies be developed for *in vivo* analysis of spatiotemporal sulfide flux?
- How does modulation of the O_2_ economy by sulfide impact physiology and behavior in hibernating and non-hibernating animals?
- How does sulfide signaling affect organellar function beyond mitochondria?
- Can sulfide oxidation products serve as a biomarker for disease progression or outcome?
- Is protein persulfidation a stochastic or regulated process with specific mechanisms for installation on distinct targets?

## Data Availability

All data are available in the manuscript.

## References

[1] Nicholson JK , Holmes E , Kinross J , Burcelin R , Gibson G , Jia W , Pettersson S . Host-gut microbiota metabolic interactions. Science. 2012;336:1262–1267. doi: 10.1126/science.1223813. 22674330

[2] Morais LH , Schreiber HLt , Mazmanian SK . The gut microbiota-brain axis in behaviour and brain disorders. Nat Rev Microbiol. 2021;19:241–255. doi: 10.1038/s41579-020-00460-0. 33093662

[3] Okumura R , Takeda K . Roles of intestinal epithelial cells in the maintenance of gut homeostasis. Exp Mol Med. 2017;49:e338–e338. doi: 10.1038/emm.2017.20. 28546564 PMC5454438

[4] Johansson ME , Larsson JM , Hansson GC . The two mucus layers of colon are organized by the MUC2 mucin, whereas the outer layer is a legislator of host-microbial interactions. Proc Natl Acad Sci U S A. 2011;108(Suppl 1):4659–4665. doi: 10.1073/pnas.1006451107. 20615996 PMC3063600

[5] Birchenough GM , Johansson ME , Gustafsson JK , Bergström JH , Hansson GC . New developments in goblet cell mucus secretion and function. Mucosal Immunol. 2015;8:712–719. doi: 10.1038/mi.2015.32. 25872481 PMC4631840

[6] McCallum G , Tropini C . The gut microbiota and its biogeography. Nat Rev Microbiol. 2024;22:105–118. doi: 10.1038/s41579-023-00969-0. 37740073

[7] McCallum G , Burckhardt JC , He J , Hong A , Potvin-Trottier L , Tropini C . A bacteroides synthetic biology toolkit to build an in vivo malabsorption biosensor. Cell. 2026;189:1245–1261 e21. doi: 10.1016/j.cell.2025.12.052. 41610848

[8] Sarfatis A , Wang Y , Twumasi-Ankrah N , Moffitt JR . Highly multiplexed spatial transcriptomics in bacteria. Science. 2025;387:eadr0932. doi: 10.1126/science.adr0932. 39847624 PMC12278067

[9] Ma P , Amemiya HM , He LL , Gandhi SJ , Nicol R , Bhattacharyya RP , Smillie CS , Hung DT . Bacterial droplet-based single-cell RNA-seq reveals antibiotic-associated heterogeneous cellular states. Cell. 2023;186:877–891 e14. doi: 10.1016/j.cell.2023.01.002. 36708705 PMC10014032

[10] Xu Z , Wang Y , Sheng K , Rosenthal R , Liu N , Hua X , Zhang T , Chen J , Song M , Lv Y , et al. Droplet-based high-throughput single microbe RNA sequencing by smrandom-seq. Nat Commun. 2023;14:5130. doi: 10.1038/s41467-023-40137-9. 37612289 PMC10447461

[11] Gaisser KD , Skloss SN , Brettner LM , Paleologu L , Roco CM , Rosenberg AB , Hirano M , DePaolo RW , Seelig G , Kuchina A . High-throughput single-cell transcriptomics of bacteria using combinatorial barcoding. Nat Protoc. 2024;19:3048–3084. doi: 10.1038/s41596-024-01007-w. 38886529 PMC11575931

[12] Schmidt F , Zimmermann J , Tanna T , Farouni R , Conway T , Macpherson AJ , Platt RJ . Noninvasive assessment of gut function using transcriptional recording sentinel cells. Science. 2022;376:eabm6038. doi: 10.1126/science.abm6038. 35549411 PMC11163514

[13] Cuevasanta E , Denicola A , Alvarez B , Möller MN , Stadler K . Solubility and permeation of hydrogen sulfide in lipid membranes. PLoS One. 2012;7:e34562. doi: 10.1371/journal.pone.0034562. 22509322 PMC3324494

[14] Attene-Ramos MS , Wagner ED , Plewa MJ , Gaskins HR . Evidence that hydrogen sulfide is a genotoxic agent. Mol Cancer Res. 2006;4:9–14. doi: 10.1158/1541-7786.MCR-05-0126. 16446402

[15] Ijssennagger N , Belzer C , Hooiveld GJ , Dekker J , van Mil SWC , Müller M , Kleerebezem M , van der Meer R . Gut microbiota facilitates dietary heme-induced epithelial hyperproliferation by opening the mucus barrier in colon. Proc Natl Acad Sci U S A. 2015;112:10038–10043. doi: 10.1073/pnas.1507645112. 26216954 PMC4538683

[16] Macfarlane GT , Gibson GR , Cummings JH . Comparison of fermentation reactions in different regions of the human colon. J Appl Bacteriol. 1992;72:57–64. 1541601 10.1111/j.1365-2672.1992.tb04882.x

[17] Florin TH . Hydrogen sulphide and total acid-volatile sulphide in faeces, determined with a direct spectrophotometric method. Clin Chim Acta. 1991;196:127–134. doi: 10.1016/0009-8981(91)90065-K. 2029779

[18] Magee EA , Richardson CJ , Hughes R , Cummings JH . Contribution of dietary protein to sulfide production in the large intestine: an in vitro and a controlled feeding study in humans. AJCN. 2000;72:1488–1494. doi: 10.1093/ajcn/72.6.1488. 11101476

[19] Jorgensen J , Mortensen PB . Hydrogen sulfide and colonic epithelial metabolism: implications for ulcerative colitis. Dig Dis Sci. 2001;46:1722–1732. doi: 10.1023/A:1010661706385. 11508674

[20] Levitt MD , Springfield J , Furne J , Koenig T , Suarez FL . Physiology of sulfide in the rat colon: use of bismuth to assess colonic sulfide production. J Appl Physiol. 2002;92:1655–1660. doi: 10.1152/japplphysiol.00907.2001. 11896034

[21] Deplancke B , Finster K , Graham WV , Collier CT , Thurmond JE , Gaskins HR . Gastrointestinal and microbial responses to sulfate-supplemented drinking water in mice. Exp Biol Med (Maywood). 2003;228:424–433. doi: 10.1177/153537020322800413. 12671187

[22] Whitfield NL , Kreimier EL , Verdial FC , Skovgaard N , Olson KR . Reappraisal of H2S/sulfide concentration in vertebrate blood and its potential significance in ischemic preconditioning and vascular signaling. Am J Physiol Regul Integr Comp Physiol. 2008;294:R1930–7. doi: 10.1152/ajpregu.00025.2008. 18417642

[23] Ishigami M , Hiraki K , Umemura K , Ogasawara Y , Ishii K , Kimura H . A source of hydrogen sulfide and a mechanism of its release in the brain. Antioxid Redox Signal. 2009;11:205–214. doi: 10.1089/ars.2008.2132. 18754702

[24] Mitsui T , Edmond LM , Magee EA , Cummings JH . The effects of bismuth, iron, zinc and nitrate on free sulfide in batch cultures seeded with fecal flora. Clin Chim Acta. 2003;335:131–135. doi: 10.1016/S0009-8981(03)00288-2. 12927694

[25] Williamson G , Engel PC , Mizzer JP , Thorpe C , Massey V . Evidence that the greening ligand in native butyryl-CoA dehydrogenase is a CoA persulfide. J Biol Chem. 1982;257:4314–4320. doi: 10.1016/S0021-9258(18)34723-9. 7068637

[26] Landry AP , Moon S , Kim H , Yadav PK , Guha A , Cho U , Banerjee R . A catalytic trisulfide in human sulfide quinone oxidoreductase catalyzes coenzyme A persulfide synthesis and inhibits butyrate oxidation. Cell Chem Biol. 2019;26:1515–1525 e4. doi: 10.1016/j.chembiol.2019.09.010. 31591036 PMC6906606

[27] Roediger WE . Role of anaerobic bacteria in the metabolic welfare of the colonic mucosa in man. Gut. 1980;21:793–798. doi: 10.1136/gut.21.9.793. 7429343 PMC1419533

[28] Ijssennagger N , van der Meer R , van Mil SWC . Sulfide as a mucus barrier-breaker in inflammatory bowel disease? Trends Mol Med. 2016;22:190–199. doi: 10.1016/j.molmed.2016.01.002. 26852376

[29] Banerjee R . Catalytic promiscuity and heme-dependent redox regulation of H_2_S synthesis. Curr Opin Chem Biol. 2017;37:115–121. doi: 10.1016/j.cbpa.2017.02.021. 28282633 PMC5410396

[30] Singh S , Banerjee R . PLP-dependent H_2_S biogenesis. Biochim Biophys Acta. 2011;1814:1518–1527. doi: 10.1016/j.bbapap.2011.02.004. 21315854 PMC3193879

[31] Blachier F , Andriamihaja M , Larraufie P , Ahn E , Lan A , Kim E . Production of hydrogen sulfide by the intestinal microbiota and epithelial cells and consequences for the colonic and rectal mucosa. Am J Physiol Gastrointest Liver Physiol. 2021;320:G125–G135. doi: 10.1152/ajpgi.00261.2020. 33084401

[32] Kumar R , Sykes DJ , Band VI , Schaller ML , Patel R , Vitvitsky V , Sajjakulnukit P , Singhal R , Wong HKA , Hourigan SK , et al. Gut sulfide metabolism modulates behavior and brain bioenergetics. Proc Natl Acad Sci U S A. 2025;122:e2503677122. doi: 10.1073/pnas.2503677122. 40526718 PMC12207524

[33] Shen X , Carlstrom M , Borniquel S , Carlström M , Jädert C , Kevil CG , Lundberg JO . Microbial regulation of host hydrogen sulfide bioavailability and metabolism. Free Radic Biol Med. 2013;60:195–200. doi: 10.1016/j.freeradbiomed.2013.02.024. 23466556 PMC4077044

[34] Hildebrandt TM , Grieshaber MK . Three enzymatic activities catalyze the oxidation of sulfide to thiosulfate in mammalian and invertebrate mitochondria. FEBS J. 2008;275:3352–3361. doi: 10.1111/j.1742-4658.2008.06482.x. 18494801

[35] Bouillaud F , Blachier F . Mitochondria and sulfide: a very old story of poisoning, feeding and signaling? Antioxid Redox Signal. 2011;15:379–391. doi: 10.1089/ars.2010.3678. 21028947

[36] Landry AP , Ballou DP , Banerjee R . Hydrogen sulfide oxidation by sulfide quinone oxidoreductase. ChemBioChem. 2021;22:949–960. doi: 10.1002/cbic.202000661. 33080111 PMC7969369

[37] Kabil O , Banerjee R . The redox biochemistry of hydrogen sulfide. J Biol Chem. 2010;285:21903–21907. doi: 10.1074/jbc.R110.128363. 20448039 PMC2903356

[38] Mishanina TV , Yadav PK , Ballou DP , Banerjee R . Transient kinetic analysis of hydrogen sulfide oxidation catalyzed by human sulfide quinone oxidoreductase. J Biol Chem. 2015;290:25072–25080. doi: 10.1074/jbc.M115.682369. 26318450 PMC4599011

[39] Landry AP , Ballou DP , Banerjee R . H_2_S oxidation by nanodisc-embedded human sulfide quinone oxidoreductase. J Biol Chem. 2017;292:11641–11649. doi: 10.1074/jbc.M117.788547. 28512131 PMC5512061

[40] Goubern M , Andriamihaja M , Nubel T , Nübel T , Blachier F , Bouillaud F . Sulfide, the first inorganic substrate for human cells. FASEB J. 2007;21:1699–1706. doi: 10.1096/fj.06-7407com. 17314140

[41] Landry AP , Moon S , Bonanata J , Cho US , Coitiño EL , Banerjee R . Dismantling and rebuilding the trisulfide cofactor demonstrates its essential role in human sulfide quinone oxidoreductase. J Am Chem Soc. 2020;142:14295–14306. doi: 10.1021/jacs.0c06066. 32787249 PMC7442744

[42] Roman JV , Shukla Y , Hanna DA , Mackson M , To T , Mootha V , Banerjee R . Hydrogen sulfide-dependent activation of human sulfide quinone oxidoreductase. J Biol Chem. 2025;301:110681. doi: 10.1016/j.jbc.2025.110681. 40912653 PMC12510033

[43] Landry AP , Ballou DP , Banerjee R . Modulation of catalytic promiscuity during hydrogen sulfide oxidation. ACS Chem Biol. 2018;13:1651–1658. doi: 10.1021/acschembio.8b00258. 29715001 PMC6449043

[44] Filipovic MR , Zivanovic J , Alvarez B , Banerjee R . Chemical biology of H_2_S signaling through persulfidation. Chem Rev. 2018;118:1253–1337. doi: 10.1021/acs.chemrev.7b00205. 29112440 PMC6029264

[45] Kumar R , Vitvitsky V , Sethaudom A , Singhal R , Solanki S , Alibeckoff S , Hiraki HL , Bell HN , Andren A , Baker BM , et al. Sulfide oxidation promotes hypoxic angiogenesis and neovascularization. Nat Chem Biol. 2024;20:1294–1304. doi: 10.1038/s41589-024-01583-8. 38509349 PMC11584973

[46] Florin THJ , Neale G , Goretski S , Cummings JH . The sulfate content of foods and beverages. J Food Compos Anal. 1993;6:140–151. doi: 10.1006/jfca.1993.1016.

[47] Rey FE , Gonzalez MD , Cheng J , Wu M , Ahern PP , Gordon JI . Metabolic niche of a prominent sulfate-reducing human gut bacterium. Proc Natl Acad Sci U S A. 2013;110:13582–13587. doi: 10.1073/pnas.1312524110. 23898195 PMC3746858

[48] Roberton AM , McKenzie CG , Sharfe N , Stubbs LB . A glycosulphatase that removes sulphate from mucus glycoprotein. Biochem J. 1993;293(Pt 3):683–689. doi: 10.1042/bj2930683. 8352735 PMC1134420

[49] Hanson BT , Dimitri Kits K , Loffler J , Löffler J , Burrichter AG , Fiedler A , Denger K , Frommeyer B , Herbold CW , Rattei T , et al. Sulfoquinovose is a select nutrient of prominent bacteria and a source of hydrogen sulfide in the human gut. ISME J. 2021;15:2779–2791. doi: 10.1038/s41396-021-00968-0. 33790426 PMC8397734

[50] Wolf PG , Cowley ES , Breister A , Matatov S , Lucio L , Polak P , Ridlon JM , Gaskins HR , Anantharaman K . Diversity and distribution of sulfur metabolic genes in the human gut microbiome and their association with colorectal cancer. Microbiome. 2022;10:64. doi: 10.1186/s40168-022-01242-x. 35440042 PMC9016944

[51] Gibson GR , Macfarlane GT , Cummings JH . Occurrence of sulphate-reducing bacteria in human faeces and the relationship of dissimilatory sulphate reduction to methanogenesis in the large gut. J Appl Bacteriol. 1988;65:103–111. doi: 10.1111/j.1365-2672.1988.tb01498.x. 3204069

[52] Plugge CM , Zhang W , Scholten JC , Stams AJM . Metabolic flexibility of sulfate-reducing bacteria. Front Microbiol. 2011;2:81. doi: 10.3389/fmicb.2011.00081. 21734907 PMC3119409

[53] Santos AA , Venceslau SS , Grein F , Leavitt WD , Dahl C , Johnston DT , Pereira IAC . A protein trisulfide couples dissimilatory sulfate reduction to energy conservation. Science. 2015;350:1541–1545. doi: 10.1126/science.aad3558. 26680199

[54] Luo W , Zhao M , Dwidar M , Gao Y , Xiang L , Wu X , Medema MH , Xu S , Li X , Schäfer H , et al. Microbial assimilatory sulfate reduction-mediated H(2)S: an overlooked role in Crohn's disease development. Microbiome. 2024;12:152. doi: 10.1186/s40168-024-01873-2. 39152482 PMC11328384

[55] Kushkevych I , Cejnar J , Treml J , Dordević D , Kollar P , Vítězová M . Recent advances in metabolic pathways of sulfate reduction in intestinal bacteria. Cells. 2020;9:698. doi: 10.3390/cells9030698. 32178484 PMC7140700

[56] Crane BR , Getzoff ED . The relationship between structure and function for the sulfite reductases. Curr Opin Struct Biol. 1996;6:744–756. doi: 10.1016/S0959-440X(96)80003-0. 8994874

[57] Anantharaman K , Hausmann B , Jungbluth SP , Kantor RS , Lavy A , Warren LA , Rappé MS , Pester M , Loy A , Thomas BC , et al. Expanded diversity of microbial groups that shape the dissimilatory sulfur cycle. ISME J. 2018;12:1715–1728. doi: 10.1038/s41396-018-0078-0. 29467397 PMC6018805

[58] Black KA , Dos Santos PC . Shared-intermediates in the biosynthesis of thio-cofactors: mechanism and functions of cysteine desulfurases and sulfur acceptors. Biochim Biophys Acta. 2015;1853:1470–1480. doi: 10.1016/j.bbamcr.2014.10.018. 25447671

[59] Braccia DJ , Jiang X , Pop M , Hall AB . The capacity to produce hydrogen sulfide (H(2)S) via cysteine degradation is ubiquitous in the human gut microbiome. Front Microbiol. 2021;12:705583. doi: 10.3389/fmicb.2021.705583. 34745023 PMC8564485

[60] Chand D , Avinash VS , Yadav Y , Pundle AV , Suresh CG , Ramasamy S . Molecular features of bile salt hydrolases and relevance in human health. Biochim Biophys Acta Gen Subj. 2017;1861:2981–2991. doi: 10.1016/j.bbagen.2016.09.024. 27681686

[61] Laue H , Cook AM . Biochemical and molecular characterization of taurine:pyruvate aminotransferase from the anaerobe bilophila wadsworthia. Eur J Biochem. 2000;267:6841–6848. doi: 10.1046/j.1432-1033.2000.01782.x. 11082195

[62] Carbonero F , Benefiel AC , Alizadeh-Ghamsari AH , Gaskins HR . Microbial pathways in colonic sulfur metabolism and links with health and disease. Front Physiol. 2012;3:448. doi: 10.3389/fphys.2012.00448. 23226130 PMC3508456

[63] Peck SC , Denger K , Burrichter A , Irwin SM , Balskus EP , Schleheck D . A glycyl radical enzyme enables hydrogen sulfide production by the human intestinal bacterium bilophila wadsworthia. Proc Natl Acad Sci U S A. 2019;116:3171–3176. doi: 10.1073/pnas.1815661116. 30718429 PMC6386719

[64] Benson AA , Daniel H , Wiser R . A sulfolipid in plants. Proc Natl Acad Sci U S A. 1959;45:1582–1587. doi: 10.1073/pnas.45.11.1582. 16590547 PMC222763

[65] Denger K , Weiss M , Felux AK , Schneider A , Mayer C , Spiteller D , Huhn T , Cook AM , Schleheck D . Sulphoglycolysis in escherichia coli K-12 closes a gap in the biogeochemical sulphur cycle. Natur. 2014;507:114–117. doi: 10.1038/nature12947. 24463506

[66] Liu JY , Wei YF , Ma KL , An J , Ang EL , Zhao H , Zhang Y . Mechanistically diverse pathways for sulfoquinovose degradation in bacteria. ACS Catal. 2021;11:14740–14750. doi: 10.1021/acscatal.1c04321.

[67] Krasenbrink J , Hanson BT , Weiss AS , Borusak S , Tanabe TS , Lang M , Aichinger G , Hausmann B , Berry D , Richter A , et al. Sulfoquinovose is exclusively metabolized by the gut microbiota and degraded differently in mice and humans. Microbiome. 2025;13:184. doi: 10.1186/s40168-025-02175-x. 40775374 PMC12330013

[68] Sharma M , Lingford JP , Petricevic M , Snow AJ , Zhang Y , Järvå MA , Mui JW , Scott NE , Saunders EC , Mao R , et al. Oxidative desulfurization pathway for complete catabolism of sulfoquinovose by bacteria. Proc Natl Acad Sci U S A. 2022;119 10.1073/pnas.e2116022119. PMC8795539 35074914

[69] Singhal R , Shah YM . Oxygen battle in the gut: hypoxia and hypoxia-inducible factors in metabolic and inflammatory responses in the intestine. J Biol Chem. 2020;295:10493–10505. doi: 10.1074/jbc.REV120.011188. 32503843 PMC7383395

[70] Byndloss MX , Olsan EE , Rivera-Chavez F , Rivera-Chávez F , Tiffany CR , Cevallos SA , Lokken KL , Torres TP , Faber F , Gao Y , et al. Microbiota-activated PPAR-gamma signaling inhibits dysbiotic enterobacteriaceae expansion. Science. 2017;357:570–575. doi: 10.1126/science.aam9949. 28798125 PMC5642957

[71] Kelly CJ , Zheng L , Campbell EL , Saeedi B , Scholz CC , Bayless AJ , Wilson KE , Glover LE , Kominsky DJ , Magnuson A , et al. Crosstalk between microbiota-derived short-chain fatty acids and intestinal epithelial HIF augments tissue barrier function. Cell Host Microbe. 2015;17:662–671. doi: 10.1016/j.chom.2015.03.005. 25865369 PMC4433427

[72] Rivera-Chavez F , Zhang LF , Faber F , Rivera-Chávez F , Lopez CA , Byndloss MX , Olsan EE , Xu G , Velazquez EM , Lebrilla CB , et al. Depletion of butyrate-producing clostridia from the gut microbiota drives an aerobic luminal expansion of salmonella. Cell Host Microbe. 2016;19:443–454. doi: 10.1016/j.chom.2016.03.004. 27078066 PMC4832419

[73] Litvak Y , Byndloss MX , Tsolis RM , Bäumler AJ . Dysbiotic proteobacteria expansion: a microbial signature of epithelial dysfunction. Curr Opin Microbiol. 2017;39:1–6. doi: 10.1016/j.mib.2017.07.003. 28783509

[74] Litvak Y , Byndloss MX , Baumler AJ . Colonocyte metabolism shapes the gut microbiota. Science. 2018;362. doi: 10.1126/science.eaat9076. PMC6296223 30498100

[75] Brake J , Banerjee R . Power and poison: the intersections of H_2_S and O_2_ metabolism. J Biol Chem. 2025;301:110810. doi: 10.1016/j.jbc.2025.110810. 41093074 PMC12661444

[76] Vital M , Howe AC , Tiedje JM . Revealing the bacterial butyrate synthesis pathways by analyzing (meta)genomic data. mBio. 2014;5:e00889. doi: 10.1128/mBio.00889-14. 24757212 PMC3994512

[77] Louis P , Flint HJ . Diversity, metabolism and microbial ecology of butyrate-producing bacteria from the human large intestine. FEMS Microbiol Lett. 2009;294:1–8. doi: 10.1111/j.1574-6968.2009.01514.x. 19222573

[78] Kaiko GE , Ryu SH , Koues OI , Collins PL , Solnica-Krezel L , Pearce EJ , Oltz EM , Stappenbeck TS . The colonic crypt protects stem cells from microbiota-derived metabolites. Cell. 2016;165:1708–1720. doi: 10.1016/j.cell.2016.05.018. 27264604 PMC5026192

[79] Fan YY , Davidson LA , Callaway ES , Wright GA , Safe S , Chapkin RS . A bioassay to measure energy metabolism in mouse colonic crypts, organoids, and sorted stem cells. Am J Physiol Gastrointest Liver Physiol. 2015;309:G1–9. doi: 10.1152/ajpgi.00052.2015. 25977509 PMC4491508

[80] Lee JY , Cevallos SA , Byndloss MX , Tiffany CR , Olsan EE , Butler BP , Young BM , Rogers AW , Nguyen H , Kim K , et al. High-fat diet and antibiotics cooperatively impair mitochondrial bioenergetics to trigger dysbiosis that exacerbates pre-inflammatory bowel disease. Cell Host Microbe. 2020;28:273–284 e6. doi: 10.1016/j.chom.2020.06.001. 32668218 PMC7429289

[81] Litvak Y , Mon KKZ , Nguyen H , Chanthavixay G , Liou M , Velazquez EM , Kutter L , Alcantara MA , Byndloss MX , Tiffany CR , et al. Commensal enterobacteriaceae protect against salmonella colonization through oxygen competition. Cell Host Microbe. 2019;25:128–139 e5. doi: 10.1016/j.chom.2018.12.003. 30629913 PMC12036633

[82] Savage HP , Bays DJ , Tiffany CR , Gonzalez MA , Bejarano EJ , Carvalho TP , Luo Z , Masson HL , Nguyen H , Santos RL , et al. Epithelial hypoxia maintains colonization resistance against candida albicans. Cell Host Microbe. 2024;32:1103–1113 e6. doi: 10.1016/j.chom.2024.05.008. 38838675 PMC11239274

[83] Larabi A.B , Bejarano E.J , de Carvalho T.P , Masson H.L.P , Nguyen H. , Radlinski L.C , Yansane N. , Mahan S.P , Kumar R. , Shah Y.M , et al. Sulfide detoxification drives epithelial hypoxia and microbiota homeostasis in the gut. 2026. submitted for publication.

[84] Suarez FL , Furne JK , Springfield J , Levitt MD . Bismuth subsalicylate markedly decreases hydrogen sulfide release in the human colon. G. astroenterology. 1998;114:923–929. doi: 10.1016/S0016-5085(98)70311-7. 9558280

[85] Band VI , LaPoint P , Levy S , Krausfeldt L , Lacroix IS , Chong A , Brandes NT , Schwarz B , Mistry s , Burns AS , et al. Bismuth subsalicylate profoundly alters gut microbiome and immunity with increased susceptibility to infection. medRxiv. 2025. doi: 10.1101/2025.10.01.25337000.

[86] Libiad M , Vitvitsky V , Bostelaar T , Bak DW , Lee H , Sakamoto N , Fearon E , Lyssiotis CA , Weerapana E , Banerjee R . Hydrogen sulfide perturbs mitochondrial bioenergetics and triggers metabolic reprogramming in colon cells. J Biol Chem. 2019;294:12077–12090. doi: 10.1074/jbc.RA119.009442. 31213529 PMC6690701

[87] Leschelle X , Goubern M , Andriamihaja M , Blottière HM , Couplan E , Gonzalez-Barroso M , Petit C , Pagniez A , Chaumontet C , Mignotte B , et al. Adaptative metabolic response of human colonic epithelial cells to the adverse effects of the luminal compound sulfide. Biochim Biophys Acta. 2005;1725:201–212. doi: 10.1016/j.bbagen.2005.06.002. 15996823

[88] Hanna DA , Diessl J , Guha A , Kumar R , Andren A , Lyssiotis C , Banerjee R . H(2)S preconditioning induces long-lived perturbations in O(2) metabolism. Proc Natl Acad Sci U S A. 2024;121:e2319473121. doi: 10.1073/pnas.2319473121. 38478695 PMC10962982

[89] Hanna DA , Chen B , Shah YM , Khalimonchuk O , Cunniff B , Banerjee R . H_2_S remodels mitochondrial ultrastructure and destabilizes respiratory supercomplexes. J Biol Chem. 2025;301:108433. doi: 10.1016/j.jbc.2025.108433. 40120684 PMC12022479

[90] Hanna DA , Vitvitsky V , Banerjee R . A growth chamber for chronic exposure of mammalian cells to H_2_S. Anal Biochem. 2023;673:115191. doi: 10.1016/j.ab.2023.115191. 37207973 PMC10668543

[91] Kumar R , Landry AP , Guha A , Vitvitsky V , Lee HJ , Seike K , Reddy P , Lyssiotis CA , Banerjee R . A redox cycle with complex II prioritizes sulfide quinone oxidoreductase-dependent H_2_S oxidation. J Biol Chem. 2022;298:101435. doi: 10.1016/j.jbc.2021.101435. 34808207 PMC8683732

[92] Cogliati S , Frezza C , Soriano ME , Varanita T , Quintana-Cabrera R , Corrado M , Cipolat S , Costa V , Casarin A , Gomes LC , et al. Mitochondrial cristae shape determines respiratory chain supercomplexes assembly and respiratory efficiency. Cell. 2013;155:160–171. doi: 10.1016/j.cell.2013.08.032. 24055366 PMC3790458

[93] Vitvitsky V , Kumar R , Diessl J , Hanna DA , Banerjee R . Rapid HPLC method reveals dynamic shifts in coenzyme Q redox state. J Biol Chem. 2024;300:107301. doi: 10.1016/j.jbc.2024.107301. 38641068 PMC11109469

[94] Vitvitsky V , Kumar R , Libiad M , Maebius A , Landry AP , Banerjee R . The mitochondrial NADH pool is involved in hydrogen sulfide signaling and stimulation of aerobic glycolysis. J Biol Chem. 2021;296:100736. doi: 10.1016/j.jbc.2021.100736. 33933447 PMC8165552

[95] Kumar R , Banerjee R . Regulation of the redox metabolome and thiol proteome by hydrogen sulfide. Crit Rev Biochem Mol Biol. 2021;56:221–235. doi: 10.1080/10409238.2021.1893641. 33722121 PMC8136436

[96] Hanna D , Kumar R , Banerjee R . A metabolic paradigm for hydrogen sulfide signaling via electron transport chain plasticity. Antioxid Redox Signal. 2023;38:57–67. doi: 10.1089/ars.2022.0067. 35651282 PMC9885546

[97] Carballal S , Vitvitsky V , Kumar R , Hanna DA , Libiad M , Gupta A , Jones JW , Banerjee R . Hydrogen sulfide stimulates lipid biogenesis from glutamine that is dependent on the mitochondrial NAD(P)H pool. J Biol Chem. 2021;297:100950. doi: 10.1016/j.jbc.2021.100950. 34252456 PMC8342795

[98] Hanna D , Kumar R , Ji Y , Sajjakulnukit P , Lyssiotis C , Jones JW , Banerjee R , et al. Hydrogen sulfide increases lipid droplets and remodels lipid metabolism to relieve reductive stress. 2026. submitted for publication.

[99] Brake J , Hanna DA , Kumar R , Peng Q , Landry AP , Singhal R , Weerapana E , Shah YM , Banerjee R . Hydrogen sulfide increases intracellular oxygen and inhibits the HIF response. J Biol Chem. 2026;302:111151. doi: 10.1016/j.jbc.2026.111151. 41534831 PMC12925153

[100] Gao XH , Krokowski D , Guan BJ , Bederman I , Majumder M , Parisien M , Diatchenko L , Kabil O , Willard B , Banerjee R , et al. Quantitative H_2_S-mediated protein sulfhydration reveals metabolic reprogramming during the integrated stress response. eLife. 2015;4:e10067. doi: 10.7554/eLife.10067. 26595448 PMC4733038

[101] Doka E , Pader I , Biro A , Dóka É , Bíró A , Johansson K , Cheng Q , Ballagó K , Prigge JR , Pastor-Flores D , et al. A novel persulfide detection method reveals protein persulfide- and polysulfide-reducing functions of thioredoxin and glutathione systems. Sci Adv. 2016;2:e1500968. doi: 10.1126/sciadv.1500968. 26844296 PMC4737208

[102] Zivanovic J , Kouroussis E , Kohl JB , Adhikari B , Bursac B , Schott-Roux S , Petrovic D , Miljkovic JL , Thomas-Lopez D , Jung Y , et al. Selective persulfide detection reveals evolutionarily conserved antiaging effects of S-Sulfhydration. Cell Metab. 2020;30:1152–1170. doi: 10.1016/j.cmet.2019.10.007. PMC7185476 31735592

[103] Blackstone E , Morrison M , Roth MB . H_2_S induces a suspended animation-like state in mice. Science. 2005;308:518–518. doi: 10.1126/science.1108581. 15845845

[104] Blackstone E , Roth MB . Suspended animation-like state protects mice from lethal hypoxia. Shock. 2007;27:370–372. doi: 10.1097/SHK.0b013e31802e27a0. 17414418

[105] Barnes BM . Freeze avoidance in a mammal: body temperatures below 0 degree C in an Arctic hibernator. Science. 1989;244:1593–1595. doi: 10.1126/science.2740905. 2740905

[106] Friederich MW , Elias AF , Kuster A , Laugwitz L , Larson AA , Landry AP , Ellwood‐Digel L , Mirsky DM , Dimmock D , Haven J , et al. Pathogenic variants in SQOR encoding sulfide:quinone oxidoreductase are a potentially treatable cause of leigh disease. J Inherit Metab Dis. 2020;43:1024–1036. doi: 10.1002/jimd.12232. 32160317 PMC7484123

[107] Lobel L , Cao YG , Fenn K , Glickman JN , Garrett WS . Diet posttranslationally modifies the mouse gut microbial proteome to modulate renal function. Science. 2020;369:1518–1524. doi: 10.1126/science.abb3763. 32943527 PMC8178816

[108] Stummer N , Feichtinger RG , Weghuber D , Kofler B , Schneider AM . Role of hydrogen sulfide in inflammatory bowel disease. Antioxidants (Basel). 2023;12:1570. doi: 10.3390/antiox12081570. 37627565 PMC10452036

[109] Metwaly A , Dunkel A , Waldschmitt N , Raj ACD , Lagkouvardos I , Corraliza AM , Mayorgas A , Martinez-Medina M , Reiter S , Schloter M , et al. Integrated microbiota and metabolite profiles link Crohn's disease to sulfur metabolism. Nat Commun. 2020;11:4322. doi: 10.1038/s41467-020-17956-1. 32859898 PMC7456324

[110] Lin H , Yu Y , Zhu L , Lai N , Zhang L , Guo Y , Yang D , Ren N , Dong Q . Implications of hydrogen sulfide in colorectal cancer: mechanistic insights and diagnostic and therapeutic strategies. Redox Biol. 2023;59:102601. doi: 10.1016/j.redox.2023.102601. 36630819 PMC9841368

[111] Moore J , Babidge W , Millard S , Roediger W . Colonic luminal hydrogen sulfide is not elevated in ulcerative colitis. Dig Dis Sci. 1998;43:162–165. doi: 10.1023/A:1018848709769. 9508519

[112] David LA , Maurice CF , Carmody RN , Gootenberg DB , Button JE , Wolfe BE , Ling AV , Devlin AS , Varma Y , Fischbach MA , et al. Diet rapidly and reproducibly alters the human gut microbiome. Natur. 2014;505:559–563. doi: 10.1038/nature12820. PMC3957428 24336217

[113] Wang Y , Nguyen LH , Mehta RS , Song M , Huttenhower C , Chan AT . Association between the sulfur microbial diet and risk of colorectal cancer. JAMA Netw Open. 2021;4:e2134308. doi: 10.1001/jamanetworkopen.2021.34308. 34767023 PMC8590167

[114] Nguyen LH , Cao Y , Hur J , Mehta RS , Sikavi DR , Wang Y , Ma W , Wu K , Song M , Giovannucci EL , et al. The sulfur microbial diet is associated with increased risk of early-onset colorectal cancer precursors. Gastroenterology. 2021;161:1423–1432 e4. doi: 10.1053/j.gastro.2021.07.008. 34273347 PMC8545755

[115] Liu X , Wan X , Zhang L , Li Y , Ao Y , Zhuang P , Wu Y , Jiao J . The sulfur microbial diet and increased risk of obesity: findings from a population-based prospective cohort study. Clin Nutr. 2023;42:764–772. doi: 10.1016/j.clnu.2023.03.011. 37003050

[116] Deng K , Wang L , Nguyen SM , Shrubsole MJ , Cai Q , Lipworth L , Gupta DK , Zheng W , Shu X , Yu D . A dietary pattern promoting gut sulfur metabolism is associated with increased mortality and altered circulating metabolites in low-income American adults. EBioMedicine. 2025;115:105690. doi: 10.1016/j.ebiom.2025.105690. 40188743 PMC12001102

